# Research progress in self-oscillating polymer brushes

**DOI:** 10.1039/d1ra07374e

**Published:** 2022-01-06

**Authors:** Bao-ying Zhang, Hai-nan Luo, Wei Zhang, Yang Liu

**Affiliations:** School of Chemical Engineering, China University of Mining and Technology Xuzhou Jiangsu 221116 China baoying31@126.com; School of Chemistry, Chemical Engineering and Materials Science, Zaozhuang University Zaozhuang Shandong 277160 China

## Abstract

Polymer brushes possess unique changes in physical and chemical properties when they are exposed to external stimuli and have a wide range of applications. Self-oscillating polymers are anchored on surfaces of certain materials and are coupled with some self-oscillating reactions (with the Belousov–Zhabotinsky (BZ) reaction as an example) to form self-oscillating polymer brushes. As an independent field of stimulus response functional surface research, the development of new intelligent bionic materials has good potential. This article reviews the oscillation mechanisms of self-oscillating polymer brushes and their classifications. First, the oscillation mechanisms of self-oscillating polymer brushes are introduced. Second, the research progress in self-oscillating polymers is discussed in terms of the type of self-oscillation reactions. Finally, possible future developments of self-oscillating polymer brushes are prospected.

## Abstract 2

Polymer brushes possess unique changes in physical and chemical properties when they are exposed to external stimuli and have a wide range of applications.

## Graphical abstract

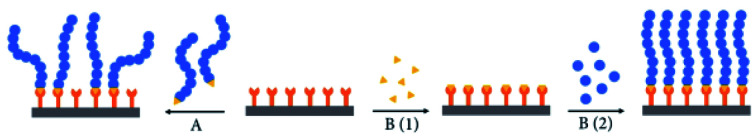

## 1. Introduction

Stimuli-responsive polymers, whose physical and chemical properties can vary in response to external stimuli including heat,^1–3^ light,^4–7^ and pH,^8–10^ have attracted much attention for their broad applications^11^ in fields such as cell surface manipulation,^12–14^ nano drivers,^15,16^ and biomolecular separation.^17,18^ Based on the density of the chains and the interactions between the chain and the interface, stimulus-responsive polymers can be categorized into single-chain polymers, multi-layer polymer components, and polymer brushes.^19^ The single-chain polymers have the lowest surface density while polymer brushes possess the highest one. Among them, polymer brushes are a focus of research interest.^19–26^

A polymer brush, defined as a thin polymer coating, is a collection of polymer chains, which are sufficiently intensive and surface-tethered, conferring a powerful tool for controlling interface properties.^24^ In particular, the repulsive interaction of chains can only be eradicated by the chain extensions perpendicular to the substrate surface.^23^ The research on polymer brushes emerged as early as the 1950s; grafting polymers onto colloidal particles was found to be a highly efficient method of preventing flocculation.^19^

**Fig. 1:**
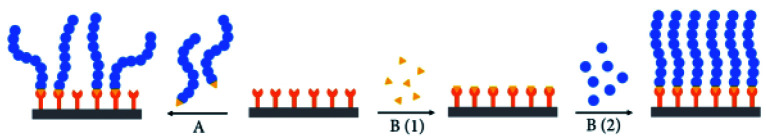
The (A) grafting-to and (B) grafting-from approaches to generate polymer films on surfaces. For (B), (1): initiator/catalyst attachment to surface, and (2): polymerization from the surface (reprinted with permission from ref. 33; copyright (2021) Qingchuan Chen).

Polymer brushes now can be immobilized on surfaces of substrates by physical adsorption or covalent chemisorption,^27–29^ while in the past, polymer brushes were attached to surfaces mainly by physical adsorption,^30^ which had insurmountable disadvantages such as thermal instability. Hence, covalent chemisorption is regarded as a preferred method for overcoming the defects of physical adsorption.^31^ Further, the method of chemisorption can be classified into two approaches, namely ‘grafting on’ and ‘grafting from’ (as illustrated in Fig. 1). For the former approach, pre-synthesized polymer chains are grafted to the surface, directly triggering the growth of polymer chains from the surface that requires to be functionalized by a monomolecular film of the initiator.^32^ However, for the preparation of polymer brushes with high graft density, the approach of ‘grafting on’ is additionally required to process the diffusion of small monomers to the surface rather than the pre-synthetization of polymer chains, thus raising the quality of transport and lessening steric hindrance.^25^ Therefore, the approach of ‘grafting from’ is more competitive since it can produce polymers with high density and thickness, which is also known as ‘surface-initiated polymerization’ (SIP).^21^

The SIP reaction confers materials with diverse properties and applications to be covalently immobilized on various kinds of substrates to shaped polymer brushes featuring different compositions and structures. A great number of types of SIP responses have been developed recently.^33,34^ The predominant one among them is the surface-initiated free radical polymerization (SI-FRP)^35^ in addition to different kinds of surface-initiated controlled radical polymerization (SI-CRP) including surface-initiated nitrogen-mediated polymerization (SI-NMP),^36^ surface-initiated atom transfer free radical polymerization (SI-ATRP),^19,37^ and surface-initiated reversible addition–fragmentation chain transfer (SI-RAFT).^38^ Especially, SI-ATRP can be seen as an efficient way to fabricate polymer brushes with a high-density structure on substrates.^39–41^

The response of the polymer brushes to the outside world is unidirectional, which means that the repeated on–off of the external stimulus is requisite for stimulating the bidirectional action. Consequently, its surface properties are controlled by external stimuli.^11^ This makes self-oscillating polymer brushes, of which surface properties can change spontaneously without external stimuli, enjoy wide application prospects.

Self-oscillating reactions, typified by Belousov–Zhabotinsky (BZ) reaction, were observed to be a reaction network with special space-time dynamic characteristics, being conventionally applied in bionic systems.^42,43^ In 1996, Yoshida *et al.* successfully grafted a ruthenium bipyridine complex which is a catalyst for the BZ reaction, onto the polymer network and acquired self-oscillating polymers with periodic expansion and contraction. They accomplished the coupling of polymer gel and oscillation reaction^44^ and they opened an avenue for subsequent research on self-oscillating polymer systems.^45–47^ Various self-oscillating polymers were largely reported, covering artificial muscle,^48^ self-oscillating cilia,^49^ self-propelling gel,^50^ self-jumping micro pump^51^ and other types.^52–61^

Self-oscillating polymer brushes, featuring the coupling between self-oscillating reaction and polymer brush, could oscillate independently and alter surface properties without external stimuli. Thus, the functional surface of self-oscillating polymer brushes could be more ‘smart’ compared with other types of polymer brushes, retaining significant strengths in large-scale transportation and control and providing a new direction for advancing the latest intelligent bionic materials.

Despite the ‘smart’ function of self-oscillating polymer brushes, the research interest on it is far less than that of stimulation-responsive polymer brushes.^62–69^ With a view to arousing more research interest in related aspects, this paper aims to systematically introduce the research progress of self-oscillating polymer brushes and summarizes its response mechanisms and classification. Additionally, the future prospective is also discussed.

## 2. Mechanisms of self-oscillating functional surface materials

According to the types of oscillatory reaction, self-oscillating polymer brushes could be divided into two broad categories, pH and BZ oscillatory reactions.

### 2.1 Mechanisms of pH oscillation

A typical example of the pH oscillation adopted a bromate–sulfite–ferrocyanide (BSF) oscillation, which produced periodic pH changes accompanied by continuous sample injection, normally in a pH range of 3.1–6.6.^70^

On the basis of oscillatory theories, Rábai, Kaminaga and Hanazaki (RKH) put forward a mechanically complex BSF-pH oscillation mechanisms^71^ that involve two principal processes: the oxidation of sulfite by bromine (eqn (3) and (4)) and the oxidation of ferrocyanide by bromine (eqn (5)).

1 SO_3_^2−^ + H^+^ ⇌ HSO_3_^−^

2 HSO_3_^−^ + H^+^ ⇋ H_2_SO_3_

3 BrO_3_^−^ + 3HSO_3_^−^ → Br^−^ + 3SO_4_^2−^ + 3H^+^

4 BrO_3_^−^ + H_2_SO_3_ → Br^−^ + 3SO_4_^2−^ + 6H^+^

5 BrO_3_^−^ + 6Fe(CN)_6_^4−^ + 6H^+^ → Br^−^ + 6Fe(CN)_6_^3−^ + 3H_2_O

When sufficient concentration of SO_3_^2−^ were input, reactions (1) and (3) dominated, converting a weak acid HSO_3_^−^ into a strong, fully ionized acid HSO_4_^−^ and producing H^+^. When the concentration of SO_3_^2−^ was sufficiently reduced by its consumption, a great amount of H^+^ was produced to generate H_2_SO_3_ by reaction (2). By reaction (4), H_2_SO_3_ was consumed to form H^+^ and other substances were consumed by reaction (5). In this case, reaction (1) and (3) were positive feedback; reactions (2) and (4) were autocatalytic production of H^+^, while reaction (5) refers to another negative feedback continuously consuming H^+^. The alternation of positive and negative feedback created pH oscillations in the system.

Nevertheless, the periodic change of pH required continuous sample injection, which limited the application of the pH oscillation system.

### 2.2 BZ reaction self-oscillation mechanisms

The mechanisms of the BZ reaction was highly complex. The classic mechanisms was proposed by Field, Koro and Noyes (FKN mechanisms),^72^ generally consisting of three steps that correspond to the consumption of Br^−^, the autocatalysis of HBrO_2_, and the creation of Br^−^ respectively. These three key substances, which were primarily responsible for activating and inhibiting the feedback loops, competed with each other at different stages of the reaction, thus periodically altering the concentration of the reaction intermediates. The reaction equations are as follows.

6 BrO_3_^−^ + 5Br^−^ + 6H^+^ → 3Br_2_ + 3H_2_O

7 BrO_3_^−^ + HBrO_2_ + 2M_red_ + 3H^+^ → 2HBrO_2_ + M_ox_ + H_2_O

8 2M_ox_ + MA + BrMA → 2M_red_ + *f*Br^−^ + other products

In these equations, the MA, M_red,_ M_ox,_ and BrMA referred to malonic acid, its reduced and oxidized states, and bromomalonic acid, respectively. The oscillation was induced by the interplay of positive and negative feedback in the system. In reaction (6), when the concentration of Br^−^ was consumed below a certain critical level, the autocatalysis of HBrO_2_ started to dominate by reaction (7), and in reaction (8) the catalyst was reduced. Then Br^−^ was produced again and MA was brominated. The character ‘*f*’ referred to the measurement coefficient of Br^−^, demonstrating the number of Br^−^ released by the reduction of each catalyst, which relied on the relative concentration of organic matter and catalyst.

Under the FKN model, as an inhibitor of oscillation, Br^−^ kept the system in a restored state. On the contrary, substances including HBrO_2_ and catalysts could function as activators and facilitate oscillation. In each period of oscillation, the BZ reaction generated intermediates of several chemical reactions, with different physicochemical properties in terms of charge, hydrophobicity, *etc.* Meanwhile, more substances such as Br_2_O_4_, BrO_2_^−^ were present to promote oscillations while the others, such as Br_2,_ emerged to restrain oscillations.^43^

### 2.3 Self-oscillation polymer brushes oscillation mechanisms

With the help of controlled free-radical polymerization, such as SI-ATRP, the ends of the self-oscillating polymer chain^73,74^ could be well immobilized on the surface.^75^ However, spatial constraints, interaction between polymer chains, dynamic balance between elastic free energy and the polymer chain was forced to stretch and extend perpendicular to the grafting position, thereby decreasing the concentration of polymer chain in the layer and enhancing the thickness of the layer.^19,76^ When coupling to external stimuli, the polymer brushes responded by hydrophilic and hydrophobic interactions, caucusing the their volume change to induce mechanical work.^77^ Subsequently, the conversion of chemical and mechanical energy took place spontaneously to form the motion^78^ that ‘passed’ along the chain, creating the shift of the polymer chain from extension to collapse or contraction.^22^

## 3. pH-responsive self-oscillating polymer brushes

**Fig. 2:**
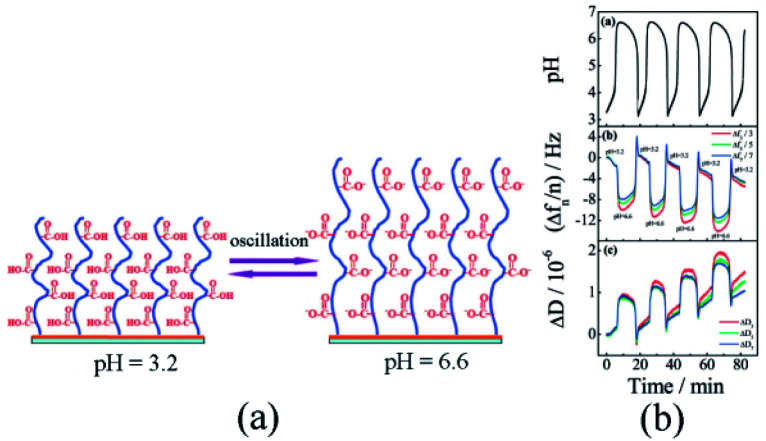
(a) Schematic diagram of pH-responsive poly(acrylic) brush. (b) Changes in polymer brush frequency and dissipation caused by pH oscillations (reprinted with permission from ref. 79; copyright (2008) American Chemical Society).

In 2008, Liu and Zhang^79^ first published their research work on self-oscillating polymer brushes. In this work, the piranha solution was utilized to clean the gold-plated resonators. Using persistent nitrogen purge, a resonator with one side protected by a Teflon shell was placed in the anhydrous ethanol solution (1.0 mM HS(CH_2_)_11_OOC(CH_3_)_2_CBr) and the single-layer initiator modified resonator was prepared and immediately applied for polymerization experiments. Sodium acrylate and 2,2′-bipyridine were dissolved in water and deoxidated through four freeze–thaw cycles. Under the protection of N_2_, CuBr and CuBr_2_ were added and stirred for ten-minute at 25 °C. In an initiator-modified resonator, the poly sodium acrylate (PSA) brush (Fig. 2a) was acquired through a 22 hours reaction incited by SI-ATRP.

The prepared poly(acrylic acid) brush was further placed in the mixture of continuous BSF reactions to detect periodic conformational changes. As the frequency and dissipation changes measured by a quartz crystal microbalance with dissipation (QCM-D), periodic expansion and contraction of the polymer brush were certified. In addition to changes in its thickness, viscosity and elastic modulus, the QCM-D results indicated that surface oscillations occurred (Fig. 2b) due to the pH oscillation. In the system, the polymer chain was induced only by pH stimuli from the environment and did not proactively participate in oscillations. Considering the wide application of solid–liquid interfaces in living and industrial systems, the self-oscillating polymer brush could be adopted in fields as artificial organs, water pollution prevention technologies and nanodevices.

In a pH-responsive self-oscillating system, there was a passive response whereby the polymer only responds to the oscillation caused by the system. In the meantime, the oscillating system required continuous injection to retain periodic pH changes in order that the polymer could oscillate persistently.

## 4. BZ reaction self-oscillating polymer brushes

Masuda's research group was committed to studying the BZ reactive self-oscillating polymer brush, with research areas as follows: the propagation of the chemical wave on the surface of self-oscillating polymer brush; the spatiotemporal action of self-initiated polymer brush oscillation; artificial cilia movement; and the influence of solidification of catalyst on the self-oscillating polymer brush.

**Fig. 3:**
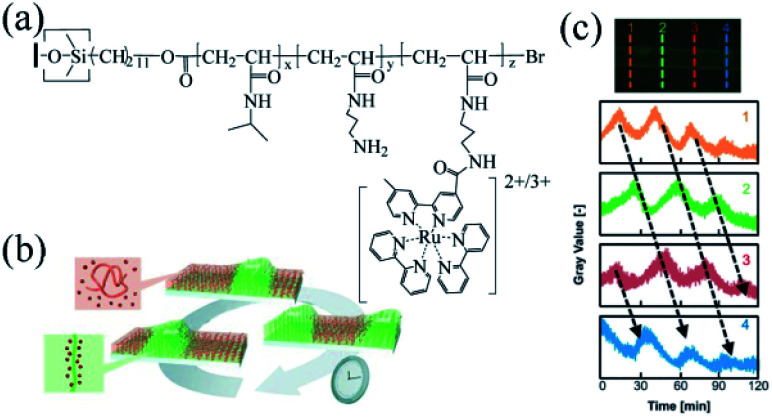
Self-oscillating polymer brushes prepared by surface-initiated atom transfer radical polymerization (SI-ATRP). (a) The chemical structure of the self-excited polymer brush^80^ (reprinted with permission from ref. 80; copyright (2013) Wiley). (b) Schematic diagram of chemical wave propagation in a polymer brush.^75^ (c) The oscillation curve of the fluorescence intensity at each position of the self-oscillating polymer brush on the inner surface of the glass capillary. [HNO_3_] = 0.81 M, [NaBrO_3_] = 0.15 M, [MA] = 0.1 M (reprinted with permission from ref. 75; copyright (2015) American Chemical Society).

In 2013, Masuda's group designed a grafting self-oscillating polymer brush. They employed the *N*-isopropylacrylamide (NIPAAm) and *N*-3-(aminopropyl)methacrylamide (NAPMAm) as the raw materials and ethyl-2-bromoisobutyrate as the initiator to synthesize the poly(NIPAAm-*co*-NAPMAm). Through the technique of SI-ATRP, they introduced bis(2,2′-bipyridine) (1-(4′-methyl-2,2′-bipyridine-4-carbonyloxy)-2,5-pyrrolidine-dione) ruthenium(ii) bis(hexafluorophosphonate) (Ru(bpy)_2_(bpy-OSu)) with succinimide group into the polymer by amino reaction with NAPMAm. Consequently, the self-oscillating polymer brush based on the BZ reaction was prepared, namely the polymer brush (NIPAAm-*co*-NAPMAm-*co*-[Ru(bpy)_3_]-NAPMAm)^80^ (Fig. 3a). Because of its photosensitivity and sensitivity to fluorescence, the self-oscillating behaviors of Ru complexes could be detected by the fluorescence intensity (Fig. 3c). The results showed that the BZ reaction occurred on the surface of the self-oscillating polymer brush and chemical waves propagated through the polymer brush layer on the inner surface of the glass capillary. The gel subjected to the mechanical swelling–deswelling oscillation at a constant temperature. It was the first autonomous functional surface consisting of synthetic polymers since oscillating gels were first reported in 1996. The research suggested that polymer brush works as a new type of autonomous functional surface, brightening potential prospects in nanoscale transmission systems.

In the previous report, the autonomous propagation of chemical waves on self-oscillating polymer brushes prepared by SI-ATRP was well studied. Nevertheless, the shortages of the scheme were the extremely low frequency and gradually decay of oscillations. Therefore, to improve the self-oscillatory behavior of polymer brush and to realize stable oscillation, further exploration of the relationship between the nanostructures on the surface and oscillation behaviors was necessary, which can instruct the optimal design of polymer brushes. In 2015, Masuda *et al.* investigated the spatiotemporal self-initiated oscillation of polymer brush. Chemical waves propagated within it which could be explained by the reaction-diffusion mechanisms.^75^ The self-oscillating polymer could form an oxidation zone where the autonomous propagation of pulse waves were activated (Fig. 3b). As the thickness of the polymer brush layer (30–100 nm) was far smaller than the size of the bulk gel (100–1000 μm), the observed duration of the pulse wave was less than that of the usual responsive gel system. Moreover, the research showed that the proper conditions of BZ reaction should be chosen, while the concentration of ruthenium catalyst immobilized on the polymer brush was also critical to induce stable oscillation of the polymer. Several physical and chemical parameters for controlling oscillatory behaviors, including the rate constant of autocatalytic reaction, the diffusion constant of activator and the activation energy of reaction and diffusion, were theoretically studied, offering guidelines for the design of the self-functional polymer brush system in the future.

**Fig. 4:**
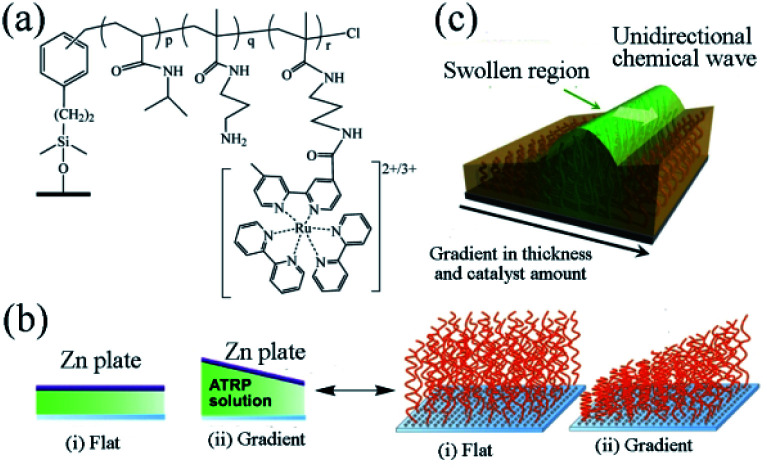
(a) The chemical structure of an oscillating polymer. (b) Preparation of flat and gradient self-oscillating polymer brushes by sacrificial anode ATRP. (c) Schematic diagram of self-oscillating gradient polymer brush, showing the unidirectional propagation of chemical waves generated by the BZ reaction^81^ (reprinted with permission from ref. 81; copyright (2016) American Association for the Advancement of Science).

In 2016, the group designed a gradient self-oscillating polymer brush surface with ordered, autonomous and unidirectional ciliary movement.^81^ The synthesis method is as follows. The initiator ((chloromethyl)phenylethyl)trimethoxysilane (ClMPETMS) was immobilized on the surface of the glass substrate by silane coupling reaction. Then the NIPAAm and NAPMAm were dissolved in DMF/water (1 : 1) mixture. Then, CuCl_2_ and tris(2-(*N*,*N*′-dimethylamino)ethyl) amine (Me_6_TREN) were added to the solution. Stirring for fifteen minutes, the ATRP catalyst system was obtained. The modified glass substrate with an immobilized ATRP initiator was set opposite the zinc surface, with the distance between the two plates adjusted by polydimethylsiloxane tablet. Afterwards, the prepared ATRP reaction liquid was injected into the gap between the two plates. The reaction lasts for one hour at 25 °C by virtue of sacrificial-anode ATRP (saATRP).^82^ After the reaction, the polymer brush (NIPAAm-*co*-NAPMAm-*co*-[Ru(bpy)_3_]-NAPMAm) with gradient was successfully prepared with the modified glass substrate cleaned with acetone, methanol and water, as well as subject to vacuum drying for three hours (Fig. 4a and b).

As an artificial model of cilia, the surface of the self-oscillating polymer brush can display autonomous wave propagation of the polymer chain at the nanoscale. Therefore, the system is regarded as an artificial experiment in cilia movement, in which the direction of the chemical waves is decided by two factors: the thickness of the polymer brush layer and the gradient in the amount of ruthenium catalyst. Affected by the gradient, the chemical wave resulting from the polymer brush propagates from the region with a low concentration of metal catalyst to the one with high concentration (Fig. 4c). It should be noticed that the gradual self-oscillating polymer brush surface can control the propagation of chemical waves in a one-dimensional path. There was effective distance in direction control. Considering the potential applications of self-oscillating polymer brushes, controlling the direction of the propagation of chemical waves presents a significant means. By adopting saATRP, the surface of the target polymer brush was designed with a thickness gradient, sufficiently controlling the direction of chemical wave of polymer self-oscillating brush. This functional surface could be applied to promote the spatiotemporal controlled material transport systems, further offering an innovative method for designing autonomous polymer brush surfaces with nanoscale activity.

In the previous discussion, the concentration of the ruthenium catalyst immobilized to the polymer brush was referred but was not clarified. To elucidate the effect of catalyst fixation on self-oscillating polymer brushes, the research group^83^ prepared self-oscillating polymers on the basis of previous studies in 2018. The specific method was as follows. The silane coupling reaction in toluene solution was performed to introduce the ATRP initiator (ClMPETMS) on the glass surface. With Cu/Me_6_TREN as ATRP catalyst and NIPAAm and NAPAMm as raw materials, glass substrates grafted with poly(Nipaam-*co*-NapMAM) were prepared by the means of SI-ATRP in the DMF/water mixture. Then, the glass substrate coated with poly(Nipaam-*co*-NapMAM) was reacted with DMSO solution containing Ru(bpy)_3_-NHS and trethylamine at 25 °C for four hours, with the substrate washed with DMSO and water as well as subject to three-hour vacuum drying to obtain the polymer brush (Nipaam-*co*-NAPmam-*co*-[Ru(BPY)_3_]NAPMAm).

**Fig. 5:**
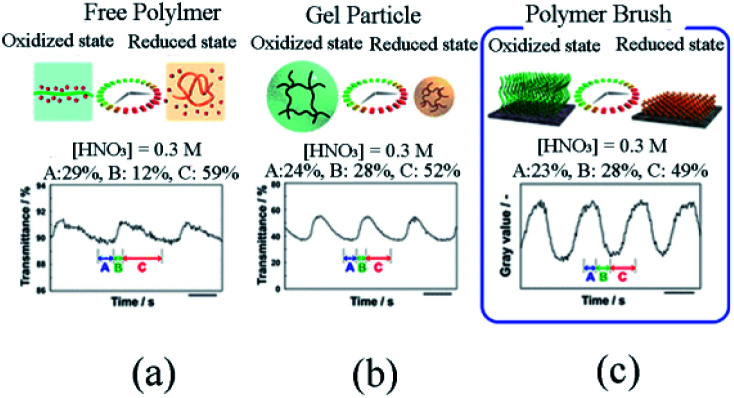
The difference in oscillation waveforms observed for (a) free polymer chains, (b) gel particles and (c) polymer brushes.^83^ The BZ reaction concentration is: [HNO_3_] = 0.3 M, [NaBrO_3_] = 0.15 M, [MA] = 0.1 M (reprinted with permission from ref. 83; copyright (2018) American Chemical Society).

Further, the self-initiated oscillation behaviors of polymer brush (Fig. 5) were explored by comparing with that of other self-initiated oscillating polymers. It was found that the initial substrate dependence of the oscillation period of polymer brush was different from that of free polymer and gel particles because the dense fixation of self-oscillating polymer on the surface restricted the contractability of Ru(bpy)_3_, leading to the disparity of initial substrate concentration dependence. In addition, based on the FKN model, the oscillation waveform of polymer brush was analyzed to supply theoretical support for comprehending polymer brush as a new reaction medium for the BZ reaction.

Homma *et al.* in Yoshida's research group studied the BZ reaction self-oscillating polymer brush from different perspectives.

In 2017, Homma *et al.*^84^ immobilized the ATRP initiator 2-(*m*/*p*-chloromethyl phenyl) ethyl trimethoxy silane (CMPEMTS) on the glass surface and then removed the immobilized initiator by maskless photolithography and oxygen plasma irradiation. On the glass coverslips, the patterned poly(NiPAAM-*co*-NapMAM) prepared by NIPAAm and *N*-3-(aminopropyl)methacrylamide (NAPMAm) was modified by SI-ATRP with the conjugation of Ru(bpy)_3_, thus generating the self-oscillating polymer brush with a pentagonal pattern.

**Fig. 6:**
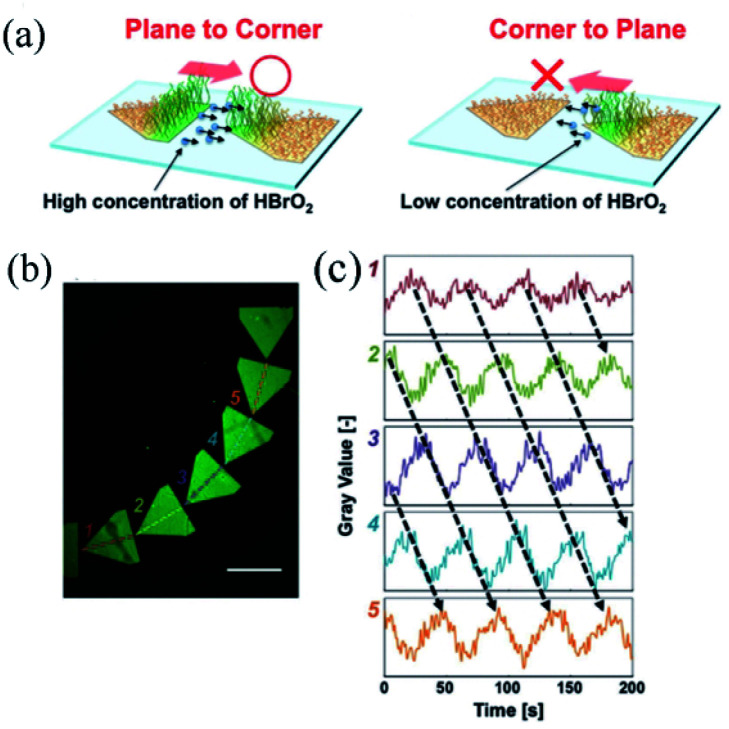
(a) Schematic diagram of the selective diffusion of chemical information HBrO_2_, accounting for the regular propagation in the oscillating space. (b) Fluorescence image of a curved array of pentagons of self-excited polymer brushes, and (c) oscillation profile of fluorescence intensity at each pentagon pattern.^84^ The scale bar is 1 mm (reprinted with permission from ref. 84; copyright (2017) Wiley).

This type of self-oscillating polymer brush was prepared by the integration of maskless photolithography and SI-ATRP technique to unidirectionally control the BZ waves. The unidirectional propagation along the pentagonal array was achieved by accurately controlling the non-reactive gap distance between pentagonal arrays. The results suggested that the ‘chemical information’^85^ of HBrO_2_ could be transmitted unidirectionally from the plane of the pattern to the corner of the adjacent one (Fig. 6a) at a proper distance. Thus inducing the control of chemical wave propagation in nanoscale self-oscillating polymer brushes, which was in line with the outcomes previously reported by Agladze *et al.*^86^ It was observed that the amount of diffused HBrO_2_ (BZ activator) was determinant in identifying the appropriate gap distance for chemical wave control. The self-oscillating polymer brush was patterned to enhance one-way control over the direction of oscillation, which could be realized in a curved array (Fig. 6b and c). Moreover, the propagation direction of chemical waves could be controlled both in a one-dimensional graph and in two dimensions. Therefore, the patterned self-oscillating polymer brush constituted a novel method for creating autonomous dynamic soft surfaces that could be used for mass transfer at the micro level.

According to the previous reports,^75,80^ autonomous redox oscillation could be induced in self-oscillating polymer brushes. It was significant to inhibit the reduced concentration of the BZ reaction intermediates (especially the activator HBrO_2_) on polymer surface and raise the amount of immobilized Ru(bpy)_3_. Based on these findings, in 2019 Homma *et al.* prepared an original oscillating polymer brush from the perspective of matrix by adopting porous glass substrate. The method of preparation is as follows. The ATRP initiator 2-(m/p-chloromethyl phenyl) ethyl trimethoxysilane (CMPETMS) was immobilized on the porous glass surface by silane coupling reaction in anhydrous toluene. With NIPAAm and NAPAMm as raw materials, glass substrates grafted with poly(Nipaam-*co*-NapMAM) were catalyzed by Cu/Me_6_TREN with SI-ATRP method in isopropyl alcohol/water. Ru(bpy)_3_-NHS and triethylamine were dissolved in DMSO and a porous glass substrate with poly(Nipaam-*co*-NAPMAM) was immobilized. After the ester group of Ru(bpy)_3_-NHS was conjugated with the amino group of NAPMAm for 24 hours at 25 °C, the modified porous glass substrate was washed with DMSO and water, then dried by vacuum for three hours to obtain the polymer brush (NIPAAm-*co*-NAPMAm-*co*-[Ru(bpy)_3_]-NAPMAm).

**Fig. 7:**
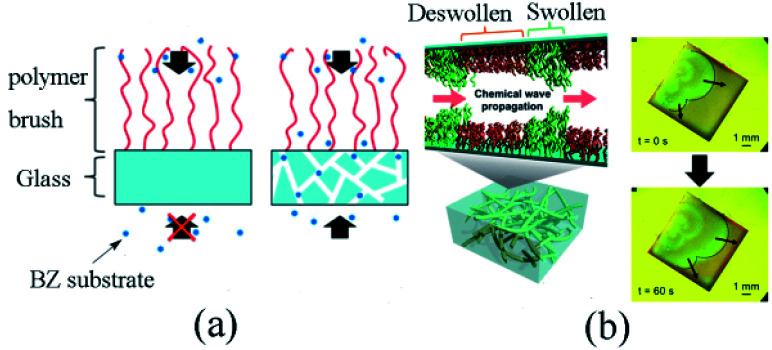
(a) Different supply mechanisms of BZ substrates for polymer brushes. For non-porous substrates, the BZ substrate can only be provided from the free end of the polymer brush, while for porous substrates, it can be effectively provided from the free end and the binding end. (b) Schematic diagram (left) and experimental observation (right) of a self-excited vibrating polymer brush made of porous glass^87^ (reprinted with permission from ref. 87; copyright (2019) American Chemical Society).

It was found that the adoption of porous glass substrate could extend the specific surface area to improve the effectivity of the metal catalyst. The polymer sustained the effective concentration of intermediate products on the surface, thus facilitated the stable oscillation of the BZ reaction process. Additionally, the BZ reactive substrate could only be supplied from the free end of the self-oscillating polymer brush on the typical glass coverslips. Conversely, the reaction substrate could also be effectively supplied from the immobilized end of the self-oscillating polymer brush (Fig. 7a) owing to the three-dimensional openness of a porous glass substrate. On the whole, the design would efficaciously induce stable self-oscillation in the polymer brush system.

Fig. 7b presents the schematic diagram and experimental observation of self-oscillating polymer brush. To understand the influence of porous media on the dynamic behaviors of self-oscillating polymer brushes, the chemical wave propagation and diffusion coefficient of self-oscillating polymer brushes were compared with that of modified ones on a traditional glass substrate. The results revealed that the wave velocity and diffusion coefficient of the porous matrix grafted from the oscillating polymer brush were remarkably lower than those of the polymer brush grown on the traditional substrate. The results suggested that the reaction-diffusion behaviors were significantly different. Furthermore, the FKN mechanisms model^72^ was also applied for the analysis of the self-oscillation, exploring the mechanisms of reaction-diffusion based on porous media. It could be seen that stable self-oscillations built on porous media delivered helpful guidance for designing the realization of mass transfer or flow control at the nanoscale.

Previous studies have demonstrated the BZ response can be modulated depending on the surface design of self-oscillating polymer brushes. Additionally, theoretical studies provided guidelines for their application. However, design strategies for inducing mechanical oscillations are yet to be explored.

In 2021, Homma, *etc.*^88^ studied the effect of the graft density on the phase transition behaviors. Their studies indicated that several critical design parameters could control the mechanical oscillation of modified polymers. Self-oscillating polymers were prepared according to the previous synthesis steps with a slight difference. ATRP initiator (CMPETMS) and its structural analogues phenylethyl trimethoxysilane (PERMS) (ATRP non-initiator) were immobilized on the glass coverslips by silane coupling reaction. At the same time, the glass coverslips with different amounts of ATRP initiator were obtained by modulating the volume ratio of ATRP initiator/non-initiator. Then the self-oscillating polymer brush (Nipaam-*co*-Napmam-Co-[Ru(bpy)_3_]-NapMAM) with controllable graft density was prepared by SI-ATRP.

**Fig. 8:**
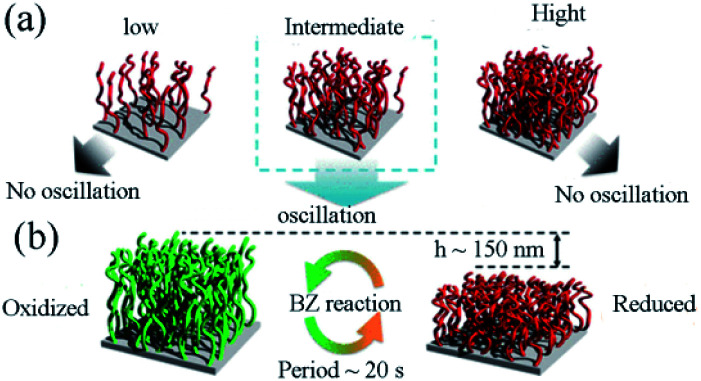
(a) Investigation of graft density effects in polymer brush. (b) Schematic illustration of the oscillatory behavior of the self-oscillating polymer brush during the BZ reaction.^88^ Concentrations of the reactants were [HNO_3_] = 0.30 M, [NaBrO_3_] = 0.15 M, and [CH_2_(COOH)_2_] = 0.10 M. *T* = 25 °C (reprinted with permission from ref. 88; copyright (2021) American Chemical Society).

The research aimed to optimize graft density to realize mechanical oscillation. The impact of density on swelling behaviors and thermal responsiveness between the redox states were significantly improved. It was found that appropriate graft density could be critical for the self-oscillation (Fig. 8a). Hence, the fine-tuning of graft density by virtue of SI-ATRP was necessary to increase the difference of swelling ratio between reduced/oxidized states. The cyclic expansion/folding motion could also be benefit from the difference of swelling ratio. In this research, digital holographic microscopy (DHM) played a vital role in precisely capturing the dynamics of polymer chains with no interference with their dynamic movement. It should be noticed that the self-initiated oscillating polymer brush the redox change was detected propagating in the form of chemical waves. The amplitude was about 150 nm with the period of twenty seconds (Fig. 8b). These records were forty times and three times higher than figures previously reported, respectively.^89^ Mechanical oscillations were achieved on a self-oscillating polymer brush prepared by SI-ATRP for the first time. It provides design guidelines for the applications in fields such as automated transport equipment.

## 5. Conclusions and prospects

Self-oscillating polymer brushes have been developed into an independent branch of stimulation-responsive polymers. In this review, the mechanisms and classification of two types of self-oscillating polymer brushes are introduced, which are expected to be applied in intelligent transmission systems in the future.

Presently, the research goal of self-oscillating polymer brushes is to achieve mass transfer or flow control at the nanoscopic scale, aiming to address two issues. The first one is related to polymerization techniques initiated by extended surfaces. So far, one of the significant limitations of the existing technical systems remains to be the need for relatively harsh reaction conditions. Factors such as low pH and strong oxidant can not only greatly affect the feasibility of the techniques applied in biological systems but also result in premature degradation and failure of the polymer system. For that reason, it is required for researchers to enrich surface-initiated polymerization techniques both chemically and topologically to break the limit and to develop biocompatible self-oscillating polymer brushes that have biomedical applications. The second issue concerns the capability to fully regulate and control the oscillation of self-oscillating polymer brushes. For this purpose, several physical and chemical parameters, including the quantity and spatial distribution of the catalyst, the rate constant of the autocatalysis reaction, the diffusion constant of the activator and the activation energy of the reaction and diffusion process, are required to be fine-tuned when the self-oscillating polymer brushes are designed and synthesized.

Currently, the future research direction of self-oscillating polymer brushes still has a great potential. The relevant innovations to be designed include fluid transport for controlling nano and microengineering systems; cilia actuators; devices capable of periodically releasing molecules or ions; equipment for controlling active sites in sensors, and soft robots. Meanwhile, the special ‘clock polymer’ can be used to modify the surface to automatically switch its performance, probably bringing a breakthrough in the advancement of the bionic autonomous soft interface. Furthermore, the available self-oscillating systems can be extended to develop new types of materials of self-oscillating polymer brushes. Overall, a large amount of research is still demanded for the ideas becoming true.

## Conflicts of interest

There are no conflicts to declare.

## Supplementary Material
